# Machado-Joseph Disease: from first descriptions to new perspectives

**DOI:** 10.1186/1750-1172-6-35

**Published:** 2011-06-02

**Authors:** Conceição Bettencourt, Manuela Lima

**Affiliations:** 1 Center of Research in Natural Resources (CIRN) and Department of Biology, University of the Azores, Ponta Delgada, Portugal; 2 Institute for Molecular and Cellular Biology (IBMC), University of Porto, Porto, Portugal; 3 Laboratorio de Biología Molecular, Instituto de Enfermedades Neurológicas de Guadalajara, Fundación Socio-Sanitaria de Castilla-La Mancha, Guadalajara, Spain

**Keywords:** Ataxin-3, *ATXN3 *gene, CAG repeats, Polyglutamine disorders, SCA3

## Abstract

Machado-Joseph Disease (MJD), also known as spinocerebellar ataxia type 3 (SCA3), represents the most common form of SCA worldwide. MJD is an autosomal dominant neurodegenerative disorder of late onset, involving predominantly the cerebellar, pyramidal, extrapyramidal, motor neuron and oculomotor systems; although sharing features with other SCAs, the identification of minor, but more specific signs, facilitates its differential diagnosis. MJD presents strong phenotypic heterogeneity, which has justified the classification of patients into three main clinical types. Main pathological lesions are observed in the spinocerebellar system, as well as in the cerebellar dentate nucleus. MJD's causative mutation consists in an expansion of an unstable CAG tract in exon 10 of the *ATXN3 *gene, located at 14q32.1. Haplotype-based studies have suggested that two main founder mutations may explain the present global distribution of the disease; the ancestral haplotype is of Asian origin, and has an estimated age of around 5,800 years, while the second mutational event has occurred about 1,400 years ago. The *ATXN3 *gene encodes for ataxin-3, which is ubiquitously expressed in neuronal and non-neuronal tissues, and, among other functions, is thought to participate in cellular protein quality control pathways. Mutated *ATXN3 *alleles consensually present about 61 to 87 CAG repeats, resulting in an expanded polyglutamine tract in ataxin-3. This altered protein gains a neurotoxic function, through yet unclear mechanisms. Clinical variability of MJD is only partially explained by the size of the CAG tract, which leaves a residual variance that should be explained by still unknown additional factors. Several genetic tests are available for MJD, and Genetic Counseling Programs have been created to better assist the affected families, namely on what concerns the possibility of pre-symptomatic testing. The main goal of this review was to bring together updated knowledge on MJD, covering several aspects from its initial descriptions and clinical presentation, through the discovery of the causative mutation, its origin and dispersion, as well as molecular genetics aspects considered essential for a better understanding of its neuropathology. Issues related with molecular testing and Genetic Counseling, as well as recent progresses and perspectives on genetic therapy, are also addressed.

## Introduction

Spinocerebellar ataxias (SCAs) are autosomal dominant inherited ataxias, which constitute a heterogeneous group of typically late-onset, progressive, and often fatal neurodegenerative disorders, characterized by progressive cerebellar dysfunction, variably associated with other symptoms of the central and peripheral nervous systems [1-3]. Nearly 30 subtypes of SCAs have been described, and based on the nature of the underlying causative mutations, these subtypes can be divided into three major categories: 1) "polyglutamine" ataxias, caused by CAG repeat expansions that encode a pure repeat of the amino acid glutamine in the corresponding protein; 2) non-coding repeat ataxias, caused by repeat expansions falling outside of the protein-coding region of the respective disease genes; and 3) ataxias caused by conventional mutations in specific genes (deletion, missense, nonsense, and splice site mutations) [1]. The focus of this review, Machado-Joseph disease (MJD; MIM #109150) [4], also known as spinocerebellar ataxia type 3 (SCA3) [5], belongs to the first of the above cited categories [6]. Several alternative designations have been given to this disorder, namely "Machado disease" [7], "nigro-spino-dentatal degeneration with nuclear ophthalmoplegia" [8], "autosomal dominant striatonigral degeneration" [9] and "Azorean disease of the nervous system" [10]. Presently, the most widely used designations are MJD and SCA3.

## Epidemiology

Globally, SCAs are considered rare disorders, with prevalence estimates varying from 0.3 to 2.0 per 100,000 [11]. MJD is presently considered the most common form of SCA worldwide [12]. The availability of a molecular test has allowed a thorough identification of cases, changing the initial geographic distribution pattern of MJD, initially thought to be related with the Portuguese discoveries and currently known to be present in many ethnic backgrounds [12], with strong geographic variation.

Among SCAs, the relative frequency of MJD is higher in countries such as Brazil (69-92%) [13,14], Portugal (58-74%) [15,16], Singapore (53%) [17], China (48-49%) [18,19], the Netherlands (44%) [11], Germany (42%) [20], and Japan (28-63%) [21,22]. It is relatively less frequent in Canada (24%) [23], United States (21%) [24], Mexico (12%) [25], Australia (12%) [26], and India (5-14%) [27,28], and it is considered as relatively rare in South Africa (4%) [29] and Italy (1%) [30].

Even within each country the geographic distribution pattern of MJD is not homogeneous. Although constituting the most prevalent subtype of SCA, in Portugal, for example, MJD is relatively rare in the mainland (1/100,000) [31], with few exceptions such as a small area of the Tagus River Valley (1/1,000) [32], but highly prevalent in the Azores Islands, where the highest worldwide prevalence occurs in Flores Island (1/239) [33].

## Clinical Presentation

MJD is a multisystem neurodegenerative disorder involving predominantly the cerebellar, pyramidal, extrapyramidal, motor neuron and oculomotor systems. A clinical diagnosis is suggested in individuals with progressive cerebellar ataxia and pyramidal signs, associated with a complex clinical picture extending from extrapyramidal signs to peripheral amyotrophy [34]. Minor, but more specific, features such as external progressive ophthalmoplegia (EPO), dystonia, intention fasciculation-like movements of facial and lingual muscles, as well as bulging eyes, may also be of major importance for the clinical diagnosis of MJD [34]. The mean age at onset is around 40 years, with extremes of 4 [35] and 70 years [31], and a mean survival time of 21 years (ranging from 7 to 29 years) [31,36]. Gait ataxia and diplopia are reported as first symptoms in 92.4% and 7.6% of cases, respectively [31].

MJD is characterized by a high degree of pleomorphism, not only in the variability in the age at onset, but also in the neurological signs presented by different patients as well as in the resulting degree of incapacity. The striking clinical heterogeneity characteristic of this disease is demonstrated by the history of its initial description. In fact, the observation of three families of Azorean ancestry (Machado, Thomas and Joseph), living in the United States of America, by three distinct groups of researchers, led to the initial description, during the 1970s, of three apparently independent diseases [7-9]. The subsequent identification of several Portuguese families living both in the Azores Islands and in the mainland of Portugal, within some of which were patients covering the three forms described, led to the unification of the disease. MJD was afterward considered as a single genetic entity, with variable phenotypic expression [4]. The marked clinical heterogeneity and the progressive nature of MJD rendered its clinical classification difficult. Coutinho and Andrade [4] systematized the disease phenotypes into three main clinical types. They observed that almost every patient presents with cerebellar signs and EPO, associated with pyramidal signs in variable degrees. Clinical types could, therefore, be distinguished on the basis of the presence/absence of important extrapyramidal signs, and the presence/absence of peripheral signs. Type 1 ("type Joseph") is characterized by an early onset (mean of 24.3 years) and a rapid progression of symptoms, which together with cerebellar ataxia and EPO include marked pyramidal and extrapyramidal signs (such as dystonia). Type 2 ("type Thomas") corresponds to presentations with an intermediate onset (mean of 40.5 years), cerebellar ataxia and EPO, with or without pyramidal sings. When present, the extrapyramidal and peripheral signs are tenuous. Patients with type 2 features may maintain these for long periods or evolve (5 to 10 years later) to type 1 or type 3, by the manifestation of important extrapyramidal or peripheral signs, respectively. Type 3 ("type Machado") presents a later onset (mean of 46.8 years) and is characterized by cerebellar ataxia and EPO, associated with peripheral alterations, with or without slight pyramidal and extrapyramidal signs [31]. As previously mentioned, these three clinical types can occasionally be present in the same family. Additionally, some authors consider as type 4 a rare presentation with parkinsonian features, with mild cerebellar deficits and a distal motor sensory neuropathy or amyotrophy [37]. Furthermore, Sakai and Kawakami [38] observed two siblings that presented spastic paraplegia without cerebellar ataxia and proposed the existence of a fifth type for MJD.

Pathological studies reveal, in most cases, that the brain weight of MJD patients is considerably reduced, in comparison to individuals without medical history of neurological or psychiatric diseases [39-42]. Furthermore, depigmentation of the substantia nigra, and atrophy of the cerebellum, pons, and medulla oblongata, as well as of the cranial nerves III to XII, has been consistently observed in MJD brains [40,43-45]. Neuropathological studies typically reveal neuronal loss in the cerebellar dentate nucleus, pons, substantia nigra, thalamus, globus pallidus, anterior horn cells and Clarke's column in the spinal cord, vestibular nucleus, many cranial motor nuclei, and other brainstem nuclei [39-41,46-55]. Such studies indicate that central nervous white matter lesions are confined to the medial lemniscus, spinocerebellar tracts and dorsal columns [39,40,45,51-55]. Although the inferior olive, as well as the cerebellar cortical neurons, were thought to be typically spared [31,41,56], conflicting results have been reported [39,40,51-53,55].

Magnetic resonance imaging (MRI) has been considered a useful tool in the study and in the diagnostic process of MJD [42,57-61]. Volumetric analyses performed on MRI of MJD patients have previously demonstrated atrophy of the cerebellum, brainstem, caudate nuclei, and putamen [62]. MR spectroscopy studies have also shown abnormalities in apparently normal deep white matter [63]. A recent study [61], using MRI-Texture analysis, showed significant differences among images texture of the caudate nucleus, thalamus, and putamen between patients and a control group, showing that this could constitute a promising tool for the detection and quantification of cerebral tissue areas affected in MJD.

## Molecular Genetics And Pathogenesis

**Figure 1:**
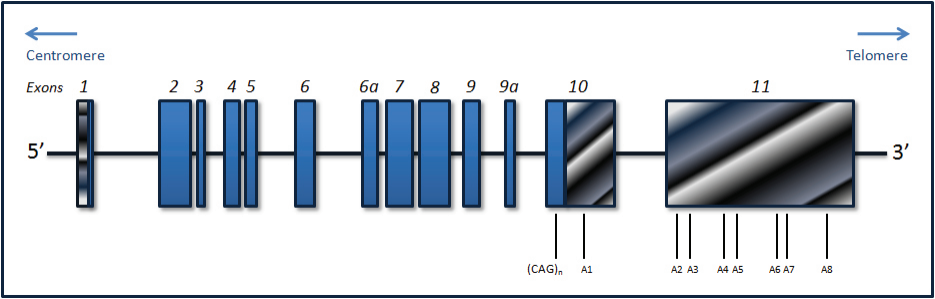
**Schematic representation of the *ATXN3 *gene structure**. Exons are numbered from 1 to 11 and are presented as boxes. Filled blue boxes indicate the coding regions, hatched horizontal boxes represent the 5'-untranslated region (UTR), and hatched diagonal boxes correspond to the 3'-UTR. The location of the polymorphic (CAG)_n _tract is indicated. Polyadenylation consensus sequences are marked from A1 to A8.

The disease locus was first mapped to the long arm of chromosome 14 (14q24.3-q32) by Takiyama *et al*. in 1993 [64]. In 1994, Kawaguchi *et al*. [65] showed that an expansion of a CAG repeat motif at the *MJD1 *gene, mapped to 14q32.1, was present in all affected individuals of a pathologically confirmed MJD family. The genomic structure of the *MJD *gene was published seven years later [66]. The gene was found to span about 48 kb and was described as containing 11 exons, with the (CAG)_n _tract located at the exon 10 (Figure 1). Two additional exons, 6a and 9a, were recently described (Figure 1) [67]. Currently, the official name of the gene is *ATXN3*, but other aliases, such as *MJD *and *MJD1*, are still in use.

Consensually, wild-type alleles range from 12 to 44 CAG repeats, whereas well established limits of expanded alleles comprise from 61 to 87 repeat units [32]. Intermediate size alleles are rare, but there are a few reports of disease associated alleles containing 56, 55, 54, 53, 51, and 45 CAG repeats [68-73]. On the other hand, an allele with 51 repeats was described, in a Portuguese family, apparently not associated with the disease [32]. Thus, there is the possibility that low penetrance alleles, of intermediate size, which are relatively frequent in other polyglutamine disorders, namely in Huntington's disease (HD) [74], may also occur in MJD.

The *ATXN3 *gene encodes for a protein named ataxin-3, which was originally reported to be composed of 339 amino acid residues plus a variable number of glutamine repeats, with an estimated molecular weight of 40-43 kDa for normal individuals [65]. Northern blot analysis showed that the *ATXN3 *mRNA is ubiquitously transcribed in neuronal and non-neuronal human tissues [66]. Moreover, such ubiquitous expression was also demonstrated, by immunohistochemical studies, at the protein level, which is expressed not only in the brain but also throughout the body, existing both in the cytoplasm and the nucleus of various cell types. However, in neurons, ataxin-3 is predominantly a cytoplasmic protein [50]. Given its ubiquitous pattern, cellular expression of the disease gene is not itself sufficient to explain selective neuronal degeneration, suggesting that other cell-specific factors are involved in the restricted neuropathology observed in MJD [50].

At least four different species of *ATXN3 *transcripts with different sizes, estimated in approximately 1.4, 1.8, 4.5, and 7.5 kb, were reported by Northern blot analysis [66]. These different mRNA species are thought to result from differential splicing of, at least, exons 10 and 11 of *ATXN3 *gene, and alternative polyadenylation of exon 11. From sequence analysis of cDNA clones, Ichikawa *et al*. [66] reported the existence of a minimum of five MJD gene products (MJD1a; pMJD1-1; pMJD2-1; pMJD5-1; H2). The MJD1a was first described by Kawaguchi *et al*. [65]. Three additional transcripts (pMJD1-1; pMJD2-1; pMJD5-1) that differ from the MJD1a, mainly at the C-terminal, were then reported by Goto *et al*. [75]. Finally, Ichikawa *et al*. [66] described the variant H2 as having an amino acid sequence identical to the one of pMJD1-1, except for a gap of 55 amino acids, which results from the skipping of exon 2 by alternative splicing. Additional *ATXN3 *splicing variants have been deposited in databases, such as ASPicDB [76]. Recently, a large number of alternative splicing variants (n = 56) generated by four types of splicing events (exon skipping, new exons, usage of alternative 5' or 3' splice sites), occurring in a simple or combined way, were described for the *ATXN3 *gene [67]. Fifty of those had not been previously described (either in the literature or in databases), and are thought to constitute new alternative splicing variants for this gene. This suggests that alternative splicing may be an important mechanism regulating ataxin-3 diversity, and clearly indicates that there are mechanisms generating variability, beyond genomic DNA.

Ataxin-3 belongs to the family of cysteine proteases. Structurally, it is composed of a globular N-terminal Josephin domain (amino acid residues 1-182 in the human protein) [77] with a papain-like fold, combined with a more flexible C-terminal tail that contains 2 or 3 ubiquitin interaction motifs (UIMs) and the polymorphic polyglutamine tract (polyQ tract) [78]. The Josephin domain (JD) contains highly conserved amino acids, reminiscent of the catalytic residues of a deubiquitinating cysteine protease. The catalytic pocket consists of a glutamine (Q9) and a cysteine (C14) residue located in the N-terminal part of JD, and of a histidine (H119) and an asparagine (N134) in the JD C-terminal part. The cysteine, the histidine, and the asparagine constitute the catalytic triad characteristic of cysteine proteases [79]. Although the physiologic role of ataxin-3 is still unclear, it has been proposed that the wild-type form acts as a deubiquitinating enzyme (DUB) in the ubiquitin-proteasome pathway [80,81]. Moreover, it has been established that ataxin-3 can be directly activated by ubiquitination [82]. Additionally, ataxin-3 has been described having a deneddylase activity [83]. Its involvement in transcriptional regulation has also been proposed [80,84]. Furthermore, the participation of ataxin-3 in the regulation of aggresome formation, as well as in the degradation of proteins sent from the endoplasmic reticulum has been described [85]. Taken together with its enzymatic properties, these facts suggest that ataxin-3 normally participates in protein quality control pathways in the cell [46,82]. Recently, it has been suggested that this protein may also be important for a correct cytoskeletal organization [86], as well as for muscle differentiation through the regulation of the integrin signaling transduction pathway [87]. In its mutated form, when the polyQ tract reaches the pathological threshold (about 50 glutamine residues), the protein is thought to gain a neurotoxic function that, as a consequence, leads to selective neuronal cell death through a not fully understood process [50,88].

From the recently described *ATXN3 *alternative splicing variants, 20 are thought to encode distinct ataxin-3 isoforms. Although by the analysis of their domain composition, it can be predicted that some may play a protective role while others may lead to increased toxicity [67], their effective role is still unknown. It also remains unexplored if differential expression of the distinct ataxin-3 isoforms could be involved in the specificity of neuronal vulnerability. Nevertheless, it has been observed that the subcellular distribution of ataxin-3 (independently of its isoform) differs in diseased brain versus normal brain. While normally it is a predominantly cytoplasmic protein in neurons (as mentioned earlier), ataxin-3 becomes concentrated in the nucleus of neurons during disease. Moreover, in many brain regions, ataxin-3 forms intranuclear inclusions [89]. These neuronal inclusions, which are also found in other polyglutamine disorders, are heavily ubiquitinated and contain certain heat shock molecular chaperones and proteasomal subunits, suggesting that they are repositories for aberrantly folded, aggregated proteins [90]. The presence of ubiquitinated neuronal intranuclear inclusions (NIIs) has thus been recognized as a neuropathologic hallmark of these diseases, although the significance of NIIs in the pathogenesis remains a matter of controversy [45]. Relatively recent neuropathologic studies [91,92] suggest that inclusions are not directly pathogenic structures and may rather be the byproduct of neuronal efforts to wall off abnormal proteins in a nontoxic manner.

## Origins And Mechanisms Of Mutation

Two large studies focus the worldwide origin of the MJD mutation [93,94]. Gaspar *et al*. [93], by haplotype analyses of three intragenic SNPs (A^669^TG/G^669^TG, C^987^GG/G^987^GG, and TAA^1118^/TAC^1118^), found that two (ACA and GGC), out of the four observed MJD haplotypes, were present in 94% of the MJD families. For the families of Azorean extraction, these two main haplotypes were found, presenting a distribution specific to the island of origin: ACA was observed in the families from Flores Island, while GGC was found in the families from São Miguel Island. These results indicated that two distinct mutational events accounted for the presence of MJD in the Azorean Islands and in families of Azorean extraction, a fact previously evidenced by studies based on the genealogical reconstruction of affected families [95,96]. In Portugal mainland, both haplotypes were also found. Worldwide, 72% of the families share the ACA, further supporting the idea of few mutational events. Based on haplotype analyses, it has been suggested that two main founder mutations may explain the present global geographic distribution of MJD [93,94]. In opposition to the Portuguese/Azorean origin that was proposed at the time of the initial descriptions of the disease, an Asian origin was recently suggested by Martins *et al*. [94]. Their work, which aimed to determine the origins, age, and spread of the two main mutational events, through more extensive haplotype analyses, revealed that the worldwide spread lineage TTACAC reaches its highest diversity in Asia (Japanese population). An ancestral STR-based haplotype was identified in that population, and a postneolithic mutation with about 5,774 ± 1,116 years old was suggested. More recent introductions of this lineage are reported for North America, Germany, France, Portugal, and Brazil. A second mutational event, in the GTGGCA lineage, is thought to be more recent (about 1,416 ± 434 years old). The matter of its origin is more controversial, but its dispersion may be mainly explained by recent Portuguese emigration [94].

The existence of repeat instability has been reported for mutated MJD alleles, similarly to what has been described for the group of "polyglutamine" disorders or for the even larger group of triplet repeat disorders, in which MJD is included [97]. However, the underlying mutational process that allows for alleles in the normal range to, ultimately, expand to pathological size is not clearly understood. Lima *et al*. [98], on a study of nearly 2,000 chromosomes of the Portuguese population, found an allelic distribution biased towards the smaller alleles, not supporting, therefore, the idea that the larger alleles could constitute a reservoir from where expanded alleles could be continuously generated. Analysis of the distribution of the CAG repeat length frequency within the four most frequent wild-type lineages (defined by intragenic polymorphisms) supports the existence of a multistep mutation mechanism on the basis of the evolution of *ATXN3 *alleles, either by gene conversion or DNA slippage [99].

## Inheritance And Genotype-Phenotype Correlations

MJD displays an autosomal dominant pattern. Therefore, each sibling of an affected individual, or an asymptomatic carrier, has an *a priori *risk of 50% of being itself a carrier, with both genders having equal probabilities of receiving/transmitting the mutated allele and expressing the disease. Very few cases (2%) of non-penetrance are known [100], and therefore, in the context of genetic counseling (GC), MJD is considered fully penetrant. However, given the fact that MJD penetrance displays an age-dependent pattern (table 1), the probability of being a mutation carrier, and consequently the *a posteriori *risk, diminishes with age in asymptomatic individuals, reaching approximately zero at the age of 70 years [33].

**Table 1:** Age-dependent risk for asymptomatic individuals with an MJD *a priori *risk of 50% (data from Bettencourt *et al*. [33]).

Age in years	Probability of detectable gene expression	Probability of heterozygous if unaffected
10	0.02	0.50

20	0.03	0.49

30	0.22	0.44

40	0.53	0.32

50	0.80	0.17

60	0.96	0.04

70	1.00	~0

An inverse correlation is found between the size of the CAG repeat tract at the expanded alleles (and consequently the size of the polyQ tract) and the age at onset of the disease. Depending on the series of patients in study, it accounts from 50% to nearly 75% of variation in the age of appearance of the first symptoms [101,102]. A similar inverse correlation has also been described at the mRNA level [103]. Furthermore, the size of the expanded alleles has also been associated with the frequency of other clinical features, such as pseudoexophthalmos and pyramidal signs, which are more frequent in subjects with larger repeats [104]. Moreover, a gene dosage effect seems to be present in MJD, since homozygosity aggravates the clinical phenotype, with a more severe progression and an early age at onset in subjects carrying the expanded allele in both chromosomes [35,105,106]. Anticipation has been reported for MJD and other triplet repeat (TR) diseases [97,107]. Such phenomenon implicates more severe phenotypes and/or earlier ages at onset in successive generations. This can be explained by the dynamic process of mutation underlying TR diseases, which involves intergenerational instability. Normal alleles are usually transmitted to the offspring without modifications [108], while most expanded alleles are unstable upon transmission due to germinal instability, especially in male meiosis [109]. The observed tendency of expanded alleles to further increase the size of its repeat tract, in successive generations, is thought to be the genetic cause of anticipation [97].

Besides the (CAG)_n _tract size, familial factors that may increase the explanation of the onset variance have been described [31,110,111]. Although the influence of environmental factors cannot be excluded, the fact that variability within families is lower than the one observed between families supports the contribution of other genetic factors, namely modifier genes, to the remaining phenotypic variance. Modifier genes of the MJD phenotype have been, so far, searched using a candidate-gene approach. Jardim *et al*. [112] analyzed the polymorphic CAG repeats in other repeat loci (SCA2, SCA6 and DRPLA), and concluded that the CAG repeat length of the larger SCA2 allele (22-23 CAG repeats) is associated with the severity of fasciculations. No associations were found with the remaining phenotypic features, namely age of onset, anticipation, and clinical types. An exhaustive search for MJD modifier genes remains difficult to perform, among other aspects, because of constrains in sample size.

## Genetic Testing And Counseling

In the early stages of the disease, when minor but specific signs are missing, when the disease seems sporadic, when it is present in patients belonging to small family units, or when the ethnic or geographic background of the patient is thought to be unusual for this disease, a clinical diagnosis of MJD may not be simple to establish. The identification of MJD's causative gene allowed the direct detection of the mutation, thus enabling the molecular diagnosis of the disease [101]. Furthermore, it allowed worldwide molecular studies about MJD, leading, as previously referred, to a distribution of cases that was clearly different from the initial scenario, obtained exclusively by clinical criteria [113]. Predictive Testing (PT) also became possible for at-risk family members, providing an accurate confirmation of the carrier/non-carrier status in asymptomatic individuals. Targeted mutation analysis of the *ATXN3 *gene is also used for the Prenatal Diagnosis (PND) of this disease [114]. However, since a positive result for the MJD mutation raises issues concerning the termination of the pregnancy, several psychological and ethic questions emerge. An alternative for PND, the Preimplantation Genetic Diagnosis (PGD) is also presently available [115]. Levels of adherence to these genetic tests remain to be determined at a large scale. In the Azores Islands, participation in PT was estimated as being around 21%. If, however, only the small Azorean island of Flores is considered, the adherence levels reach nearly 36% [116]. In another small community, the rural region of the Tagus Valley (Portugal mainland), adherence levels to PT program were also high (over 80%) [117]. These high adherence levels in small, isolated communities raise interesting issues, since in such populations genetic diseases can represent a source of stigmatization to the affected families [116]. Therefore, a careful intervention regarding genetic tests, adapted to each specific context, is mandatory.

There is a current lack of effective therapeutics for MJD (see "Patients Management"). Therefore, it is crucial to provide adequate GC to patients and their families, providing information concerning the nature of the disease, the current lack of disease treatment, the risk for other family members as well as the availability of molecular tests, previously mentioned. PT, PND and PGD are offered within the frame of a GC Program. As an example, the Portuguese GC Program, which was based mainly on the experience with HD, aims to provide to at-risk adults the access to the genetic information that can reduce the uncertainty about their genetic status. Another of its goals is to provide the necessary psychological support to allow the proper adaptation to the test results [118]. Candidates for the MJD PT Program have been defined as those: a) at 50% risk and wishing to receive genetic information; b) over 18 years old and capable of providing informed consent; c) with a molecularly confirmed familial history of MJD; and d) asymptomatic for the disease [118].

Teams offering GC to MJD families must provide adequate and comprehensible information concerning the genetics of MJD to the affected families. A study with Azorean MJD families, conducted prior to the application of the PT in this population [119] showed that a large percentage of individuals were unable to comprehend the notion of "pre-symptomatic carrier" and, therefore, could not quantify the objective risk of inheriting/transmitting the disease.

Analysis of the motives for undertaking the PT and of the impact of the test on the psychological well-being of those tested is of major importance for the design of effective GC programs. Leite *et al*. [120] developed a Psychological General Well-Being Schedule, to evaluate psychological well-being in persons coming for MJD pre-symptomatic testing in comparison with normal population. These authors observed that, contrarily to what was expected, individuals at-risk presented higher psychological well-being indicators than the control group. Two possible explanations were suggested by Leite *et al*.[120] to justify such results: a) the group of individuals at-risk has a defensive and denial attitude, and/or b) the group of individuals at-risk is psychologically more resilient, which may have motivated their adhesion to pre-symptomatic testing, through their own self-selection. Gonzalez *et al*. [116], in a short-term study of the impact of PT in the Azores, found no differences in the mean scores of depression or anxiety before and one year after the PT. These authors concluded that the disclosure of the genetic status did not decrease the psychological well-being of the individuals that undertook testing. Accordingly, the study by Rolin *et al*. [121], which compared data obtained before and 3 to 6 months after the disclosure of genetic testing results, showed no significant changes in well-being and specific distress of PT applicants, both in the individuals identified as carriers and non-carriers. A similar result to what was observed in another study in Japan [122]. Furthermore, it has been shown that the anxiety levels are reduced in those who received a non-carrier result [122,123].

With the advent of pre-symptomatic testing, several laboratory difficulties emerged, and improvements in the diagnosis of MJD had to be made. The first problem was the occurrence of intermediate size alleles, for which it is still not possible to determine whether they are associated with a phenotype or not [32]. To minimize this constrain, clinical and molecular analysis, including the determination of CAG repeat length and the establishment of intragenic haplotypes, of large pedigrees of the affected families, is essential. Furthermore, the study of the healthy population, from the same region, to assess the distribution of the normal (CAG)_n _length in that specific population, may also be important [98]. The second problem relied on the presence of homoallelism, i.e., homozygosity for two normal alleles with exactly the same (CAG)_n _length (about 10% of all test results). This was solved by studying intragenic polymorphisms, which allowed the distinction of the two normal chromosomes. Furthermore, using a new Southern blot based method, the possibility of existence of an expanded allele in the presumed homoallelic individuals can also be excluded [32]. There are limitations in sizing precision of the CAG repeats due to the existence of somatic mosaicism [124], which originates differences in (CAG)_n _length among subpopulations of lymphocytes as well as between lymphocytes (where length is usually measured) and central nervous system cells. However, for molecular diagnosis purposes, an error of ±1 CAG repeat is considered as acceptable [32].

## Patients Management And New Perspectives In Treatment

On what concerns disease treatment, effective pharmacologic approaches for the MJD treatment as well as for other SCAs are still lacking or inadequate. Symptomatic pharmacologic therapeutics are used to alleviate some of the clinical signs, namely spasticity [125,126], parkinsonism [127,128], dystonia [129,130], and muscle cramps [131]. Several clinical trials have also been carried out. The initial double-blind, placebo-controlled, clinical trials were performed with sulfamethoxazole and trimethoprim, in a small number of MJD patients [126,132-134]. From those studies, encouraging results were obtained in terms of lessened spasticity, improvements in walker-assisted gait [132], improvements in contrast sensitivity [133], mild improvements of hyperreflexia of knee jerks and of rigospasticity of the legs [134], beneficial effects on gait and coordination [126]. However, in a larger study, also double-blind and placebo-controlled, trimethoprim-sulfamethoxazole therapy showed no significant effects [135]. The treatment of MJD patients with fluoxetine, failed to improve motor abilities [136]. On the other hand, the use of taltirelin hydrate, was shown to be effective on the ataxic speech of patients with MJD [137]. The treatment with tandospirone pointed for a reduction of ataxia and of depression levels, alleviation of insomnia and leg pain, suggesting that this is a useful drug for these symptoms in patients with MJD [138]. Another trial [139] involved the clinical response of lamotrigine (LTG) on MJD patients with early truncal ataxia and the effect of LTG on the alteration of ataxin-3 expression in the transformed MJD lymphoblastoid cells. Results from this trial indicated that LTG may have significant benefits in relief of gait disturbance in MJD patients with early ataxia, which may be related to the decreased expression of mutant ataxin-3. Notwithstanding some promising results, all these trials were carried out in a small number of patients (1 to 22 patients) and over short periods of time. Studies with a length, design and sample size to provide adequate power to detect meaningful effects should be carefully planned on the basis of underlying basic science before undergoing trials [140].

In addition to pharmacological approaches, physiotherapy may help the patients to cope with the disability associated with gait problems [141]. Physical aids, such as walkers and wheelchairs, can assist the patients in their everyday activities. Moreover, regular speech therapy evaluation for dysarthria and dysphagia as well as occupational therapy may also help patients [141].

Recent advances have been made in the field of genetic therapy. The use of small interfering RNA (siRNA) has been taken as a promising approach for treating autosomal dominant disorders. Although the mouse [142] and *Caenorhabditis elegans *[143] knockout models for ataxin-3 were viable and displayed no overt phenotype, suggesting that ataxin-3 is a non-essential protein, in both cases its importance as a DUB enzyme was confirmed. Nevertheless, there is no correspondent model in humans at our days that could support the hypothesis of ataxin-3 as a non-essential protein. Therefore, discrimination between wild-type and mutant transcripts should be an important point to be addressed in therapeutics development, in order to preserve wild-type ataxin-3 expression and function. Strategies based on the presence of a single nucleotide polymorphism (SNP) have been proposed to ensure discrimination between wild-type and mutant transcripts [144]. After the understanding of the worldwide distribution of the MJD haplotypes [93,94], the intragenic SNP G^987^GG/C^987^GG at the 3' end of the CAG tract, which variant C is present in more than 70% of the expanded alleles, seemed to bring good perspectives to the possibility of discriminating between wild-type and mutant *ATXN3 *alleles. Promising results were obtained by Alves *et al*. [145], who, using siRNA assays targeting that SNP, reached therapeutic efficacy and selectivity in a rat model of MJD. However, transposing this to MJD patients would result inefficient in the case of homozygosis for the C variant, or in the absence of this variant in the expanded allele. Thus, the search for *ATXN3 *transcript variation is still imperative for the application of such siRNA approaches. Recently, another strategy for allele-specific silencing of the mutant *ATXN3 *mRNA was applied [146], via antisense oligomers, that discriminate between the wild-type and the expanded alleles on the basis of the (CAG)_n _repeat length in cell lines. Much is still needed to transpose those allele-specific silencing strategies to effective treatment of patients, but good perspectives are foreseen in the future.

## Competing interests

The authors declare that they have no competing interests.

## Authors' contributions

CB drafted the manuscript. ML revised critically the content of the manuscript. Both authors have read and gave their final approval of the version to be published.

## References

[B1] Soong BW Paulson HL Spinocerebellar ataxias: an update Curr Opin Neurol 2007 20 4 438 446 10.1097/WCO.0b013e3281fbd3dd 17620880

[B2] Carlson KM Andresen JM Orr HT Emerging pathogenic pathways in the spinocerebellar ataxias Curr Opin Genet Dev 2009 19 3 247 253 10.1016/j.gde.2009.02.009 19345087 PMC2721007

[B3] Tsuji S Onodera O Goto J Nishizawa M Sporadic ataxias in Japan--a population-based epidemiological study Cerebellum 2008 7 2 189 197 10.1007/s12311-008-0028-x 18418674

[B4] Coutinho P Andrade C Autosomal dominant system degeneration in Portuguese families of the Azores Islands. A new genetic disorder involving cerebellar, pyramidal, extrapyramidal and spinal cord motor functions Neurology 1978 28 7 703 709 566869 10.1212/wnl.28.7.703

[B5] Stevanin G Le Guern E Ravise N Chneiweiss H Durr A Cancel G Vignal A Boch AL Ruberg M Penet C A third locus for autosomal dominant cerebellar ataxia type I maps to chromosome 14q24.3-qter: evidence for the existence of a fourth locus Am J Hum Genet 1994 54 1 11 20 8279460 PMC1918062

[B6] Shao J Diamond MI Polyglutamine diseases: emerging concepts in pathogenesis and therapy Hum Mol Genet 2007 16 Spec No. 2 R115 123 17911155 10.1093/hmg/ddm213

[B7] Nakano KK Dawson DM Spence A Machado disease. A hereditary ataxia in Portuguese emigrants to Massachusetts Neurology 1972 22 1 49 55 5061839 10.1212/wnl.22.1.49

[B8] Woods BT Schaumburg HH Nigro-spino-dentatal degeneration with nuclear ophthalmoplegia. A unique and partially treatable clinico-pathological entity J Neurol Sci 1972 17 2 149 166 10.1016/0022-510X(72)90137-2 5053922

[B9] Rosenberg RN Nyhan WL Bay C Shore P Autosomal dominant striatonigral degeneration. A clinical, pathologic, and biochemical study of a new genetic disorder Neurology 1976 26 8 703 714 945867 10.1212/wnl.26.8.703

[B10] Romanul FC Fowler HL Radvany J Feldman RG Feingold M Azorean disease of the nervous system N Engl J Med 1977 296 26 1505 1508 10.1056/NEJM197706302962606 865531

[B11] van de Warrenburg BP Sinke RJ Verschuuren-Bemelmans CC Scheffer H Brunt ER Ippel PF Maat-Kievit JA Dooijes D Notermans NC Lindhout D Spinocerebellar ataxias in the Netherlands: prevalence and age at onset variance analysis Neurology 2002 58 5 702 708 11889231 10.1212/wnl.58.5.702

[B12] Schols L Bauer P Schmidt T Schulte T Riess O Autosomal dominant cerebellar ataxias: clinical features, genetics, and pathogenesis Lancet Neurol 2004 3 5 291 304 10.1016/S1474-4422(04)00737-9 15099544

[B13] Teive HA Munhoz RP Raskin S Werneck LC Spinocerebellar ataxia type 6 in Brazil Arq Neuropsiquiatr 2008 66 3B 691 694 10.1590/S0004-282X2008000500015 18949263

[B14] Jardim LB Silveira I Pereira ML Ferro A Alonso I do Ceu Moreira M Mendonca P Ferreirinha F Sequeiros J Giugliani R A survey of spinocerebellar ataxia in South Brazil - 66 new cases with Machado-Joseph disease, SCA7, SCA8, or unidentified disease-causing mutations J Neurol 2001 248 10 870 876 10.1007/s004150170072 11697524

[B15] Vale J Bugalho P Silveira I Sequeiros J Guimaraes J Coutinho P Autosomal dominant cerebellar ataxia: frequency analysis and clinical characterization of 45 families from Portugal Eur J Neurol 2010 17 1 124 8 10.1111/j.1468-1331.2009.02757.x 19659750

[B16] Silveira I Coutinho P Maciel P Gaspar C Hayes S Dias A Guimaraes J Loureiro L Sequeiros J Rouleau GA Analysis of SCA1, DRPLA, MJD, SCA2, and SCA6 CAG repeats in 48 Portuguese ataxia families Am J Med Genet 1998 81 2 134 138 10.1002/(SICI)1096-8628(19980328)81:2<134::AID-AJMG3>3.0.CO;2-W 9613852

[B17] Zhao Y Tan EK Law HY Yoon CS Wong MC Ng I Prevalence and ethnic differences of autosomal-dominant cerebellar ataxia in Singapore Clin Genet 2002 62 6 478 481 10.1034/j.1399-0004.2002.620610.x 12485197

[B18] Tang B Liu C Shen L Dai H Pan Q Jing L Ouyang S Xia J Frequency of SCA1, SCA2, SCA3/MJD, SCA6, SCA7, and DRPLA CAG trinucleotide repeat expansion in patients with hereditary spinocerebellar ataxia from Chinese kindreds Arch Neurol 2000 57 4 540 544 10.1001/archneur.57.4.540 10768629

[B19] Jiang H Tang BS Xu B Zhao GH Shen L Tang JG Li QH Xia K Frequency analysis of autosomal dominant spinocerebellar ataxias in mainland Chinese patients and clinical and molecular characterization of spinocerebellar ataxia type 6 Chin Med J (Engl) 2005 118 10 837 843 15989765

[B20] Schols L Amoiridis G Buttner T Przuntek H Epplen JT Riess O Autosomal dominant cerebellar ataxia: phenotypic differences in genetically defined subtypes? Ann Neurol 1997 42 6 924 932 10.1002/ana.410420615 9403486

[B21] Maruyama H Izumi Y Morino H Oda M Toji H Nakamura S Kawakami H Difference in disease-free survival curve and regional distribution according to subtype of spinocerebellar ataxia: a study of 1,286 Japanese patients Am J Med Genet 2002 114 5 578 583 10.1002/ajmg.10514 12116198

[B22] Shibata-Hamaguchi A Ishida C Iwasa K Yamada M Prevalence of spinocerebellar degenerations in the Hokuriku district in Japan Neuroepidemiology 2009 32 3 176 183 10.1159/000195686 19169038

[B23] Kraft S Furtado S Ranawaya R Parboosingh J Bleoo S McElligott K Bridge P Spacey S Das S Suchowersky O Adult onset spinocerebellar ataxia in a Canadian movement disorders clinic Can J Neurol Sci 2005 32 4 450 458 16408574 10.1017/s0317167100004431

[B24] Moseley ML Benzow KA Schut LJ Bird TD Gomez CM Barkhaus PE Blindauer KA Labuda M Pandolfo M Koob MD Incidence of dominant spinocerebellar and Friedreich triplet repeats among 361 ataxia families Neurology 1998 51 6 1666 1671 9855520 10.1212/wnl.51.6.1666

[B25] Alonso E Martinez-Ruano L De Biase I Mader C Ochoa A Yescas P Gutierrez R White M Ruano L Fragoso-Benitez M Distinct distribution of autosomal dominant spinocerebellar ataxia in the Mexican population Mov Disord 2007 22 7 1050 1053 10.1002/mds.21470 17427938

[B26] Storey E du Sart D Shaw JH Lorentzos P Kelly L McKinley Gardner RJ Forrest SM Biros I Nicholson GA Frequency of spinocerebellar ataxia types 1, 2, 3, 6, and 7 in Australian patients with spinocerebellar ataxia Am J Med Genet 2000 95 4 351 357 10.1002/1096-8628(20001211)95:4<351::AID-AJMG10>3.0.CO;2-R 11186889

[B27] Saleem Q Choudhry S Mukerji M Bashyam L Padma MV Chakravarthy A Maheshwari MC Jain S Brahmachari SK Molecular analysis of autosomal dominant hereditary ataxias in the Indian population: high frequency of SCA2 and evidence for a common founder mutation Hum Genet 2000 106 2 179 187 10.1007/s004390051026 10746559

[B28] Krishna N Mohan S Yashavantha BS Rammurthy A Kiran Kumar HB Mittal U Tyagi S Mukerji M Jain S Pal PK SCA 1, SCA 2 & SCA 3/MJD mutations in ataxia syndromes in southern India Indian J Med Res 2007 126 5 465 470 18160752

[B29] Bryer A Krause A Bill P Davids V Bryant D Butler J Heckmann J Ramesar R Greenberg J The hereditary adult-onset ataxias in South Africa J Neurol Sci 2003 216 1 47 54 10.1016/S0022-510X(03)00209-0 14607302

[B30] Brusco A Gellera C Cagnoli C Saluto A Castucci A Michielotto C Fetoni V Mariotti C Migone N Di Donato S Molecular genetics of hereditary spinocerebellar ataxia: mutation analysis of spinocerebellar ataxia genes and CAG/CTG repeat expansion detection in 225 Italian families Arch Neurol 2004 61 5 727 733 10.1001/archneur.61.5.727 15148151

[B31] Coutinho P Doença de Machado-Joseph: Tentativa de definição 1992 PhD Dissertation, Instituto de Ciências Biomédicas Abel Salazar, Porto

[B32] Maciel P Costa MC Ferro A Rousseau M Santos CS Gaspar C Barros J Rouleau GA Coutinho P Sequeiros J Improvement in the molecular diagnosis of Machado-Joseph disease Arch Neurol 2001 58 11 1821 1827 10.1001/archneur.58.11.1821 11708990

[B33] Bettencourt C Santos C Kay T Vasconcelos J Lima M Analysis of segregation patterns in Machado-Joseph disease pedigrees J Hum Genet 2008 53 10 920 923 10.1007/s10038-008-0330-y 18688568

[B34] Lima L Coutinho P Clinical criteria for diagnosis of Machado-Joseph disease: report of a non-Azorena Portuguese family Neurology 1980 30 3 319 322 7189034 10.1212/wnl.30.3.319

[B35] Carvalho DR La Rocque-Ferreira A Rizzo IM Imamura EU Speck-Martins CE Homozygosity enhances severity in spinocerebellar ataxia type 3 Pediatr Neurol 2008 38 4 296 299 10.1016/j.pediatrneurol.2007.12.006 18358414

[B36] Kieling C Prestes PR Saraiva-Pereira ML Jardim LB Survival estimates for patients with Machado-Joseph disease (SCA3) Clin Genet 2007 72 6 543 545 10.1111/j.1399-0004.2007.00910.x 17894834

[B37] Suite ND Sequeiros J McKhann GM Machado-Joseph disease in a Sicilian-American family J Neurogenet 1986 3 3 177 182 10.3109/01677068609106847 3734949

[B38] Sakai T Kawakami H Machado-Joseph disease: A proposal of spastic paraplegic subtype Neurology 1996 46 3 846 847 8618704

[B39] Iwabuchi K Tsuchiya K Uchihara T Yagishita S Autosomal dominant spinocerebellar degenerations. Clinical, pathological, and genetic correlations Rev Neurol (Paris) 1999 155 4 255 270 10367323

[B40] Rub U Brunt ER Deller T New insights into the pathoanatomy of spinocerebellar ataxia type 3 (Machado-Joseph disease) Curr Opin Neurol 2008 21 2 111 116 10.1097/WCO.0b013e3282f7673d 18317266

[B41] Yamada M Sato T Tsuji S Takahashi H CAG repeat disorder models and human neuropathology: similarities and differences Acta Neuropathol 2008 115 1 71 86 17786457 10.1007/s00401-007-0287-5

[B42] Horimoto Y Matsumoto M Akatsu H Kojima A Yoshida M Nokura K Yuasa H Katada E Yamamoto T Kosaka K Longitudinal study on MRI intensity changes of Machado-Joseph disease: correlation between MRI findings and neuropathological changes J Neurol 2011 in press 10.1007/s00415-011-5992-2 21416210

[B43] Rub U de Vos RA Schultz C Brunt ER Paulson H Braak H Spinocerebellar ataxia type 3 (Machado-Joseph disease): severe destruction of the lateral reticular nucleus Brain 2002 125 Pt 9 2115 2124 12183356 10.1093/brain/awf208

[B44] Rub U Brunt ER Gierga K Schultz C Paulson H de Vos RA Braak H The nucleus raphe interpositus in spinocerebellar ataxia type 3 (Machado-Joseph disease) J Chem Neuroanat 2003 25 2 115 127 10.1016/S0891-0618(02)00099-6 12663059

[B45] Yamada M Tan CF Inenaga C Tsuji S Takahashi H Sharing of polyglutamine localization by the neuronal nucleus and cytoplasm in CAG-repeat diseases Neuropathol Appl Neurobiol 2004 30 6 665 675 10.1111/j.1365-2990.2004.00583.x 15541006

[B46] Spinocerebellar Ataxia Type 3 http://www.ncbi.nlm.nih.gov/bookshelf/br.fcgi?book=gene&part=sca3

[B47] Eto K Sumi SM Bird TD McEvoy-Bush T Boehnke M Schellenberg G Family with dominantly inherited ataxia, amyotrophy, and peripheral sensory loss. Spinopontine atrophy or Machado-Joseph Azorean disease in another non-Portuguese family? Arch Neurol 1990 47 9 968 974 2396938 10.1001/archneur.1990.00530090038011

[B48] Sudarsky L Coutinho P Machado-Joseph disease Clin Neurosci 1995 3 1 17 22 7614089

[B49] Durr A Stevanin G Cancel G Duyckaerts C Abbas N Didierjean O Chneiweiss H Benomar A Lyon-Caen O Julien J Spinocerebellar ataxia 3 and Machado-Joseph disease: clinical, molecular, and neuropathological features Ann Neurol 1996 39 4 490 499 10.1002/ana.410390411 8619527

[B50] Paulson HL Das SS Crino PB Perez MK Patel SC Gotsdiner D Fischbeck KH Pittman RN Machado-Joseph disease gene product is a cytoplasmic protein widely expressed in brain Ann Neurol 1997 41 4 453 462 10.1002/ana.410410408 9124802

[B51] Robitaille Y Lopes-Cendes I Becher M Rouleau G Clark AW The neuropathology of CAG repeat diseases: review and update of genetic and molecular features Brain Pathol 1997 7 3 901 926 10.1111/j.1750-3639.1997.tb00893.x 9217975 PMC8098401

[B52] Schmidt T Landwehrmeyer GB Schmitt I Trottier Y Auburger G Laccone F Klockgether T Volpel M Epplen JT Schols L An isoform of ataxin-3 accumulates in the nucleus of neuronal cells in affected brain regions of SCA3 patients Brain Pathol 1998 8 4 669 679 9804376 10.1111/j.1750-3639.1998.tb00193.x PMC8098309

[B53] Gilman S The spinocerebellar ataxias Clin Neuropharmacol 2000 23 6 296 303 10.1097/00002826-200011000-00002 11575863

[B54] Yamada M Hayashi S Tsuji S Takahashi H Involvement of the cerebral cortex and autonomic ganglia in Machado-Joseph disease Acta Neuropathol 2001 101 2 140 144 11271368 10.1007/s004010000277

[B55] Koeppen AH The pathogenesis of spinocerebellar ataxia Cerebellum 2005 4 1 62 73 10.1080/14734220510007950 15895563

[B56] Wang YG Du J Wang JL Chen J Chen C Luo YY Xiao ZQ Jiang H Yan XX Xia K Six cases of SCA3/MJD patients that mimic hereditary spastic paraplegia in clinic J Neurol Sci 2009 285 1-2 121 4 15 10.1016/j.jns.2009.06.027 19608203

[B57] Imon Y Katayama S Kawakami H Murata Y Oka M Nakamura S A necropsied case of Machado-Joseph disease with a hyperintense signal of transverse pontine fibres on long TR sequences of magnetic resonance images J Neurol Neurosurg Psychiatry 1998 64 1 140 141 10.1136/jnnp.64.1.140 9436751 PMC2169922

[B58] Murata Y Yamaguchi S Kawakami H Imon Y Maruyama H Sakai T Kazuta T Ohtake T Nishimura M Saida T Characteristic magnetic resonance imaging findings in Machado-Joseph disease Arch Neurol 1998 55 1 33 37 10.1001/archneur.55.1.33 9443709

[B59] Yamada S Nishimiya J Nakajima T Taketazu F Linear high intensity area along the medial margin of the internal segment of the globus pallidus in Machado-Joseph disease patients J Neurol Neurosurg Psychiatry 2005 76 4 573 575 10.1136/jnnp.2004.040279 15774449 PMC1739610

[B60] Lee YC Liu CS Wu HM Wang PS Chang MH Soong BW The 'hot cross bun' sign in the patients with spinocerebellar ataxia Eur J Neurol 2009 16 4 513 516 10.1111/j.1468-1331.2008.02524.x 19187260

[B61] De Oliveira MS D'Abreu A Franca MC Jr Lopes-Cendes I Cendes F Castellano G MRI-Texture Analysis of Corpus Callosum, Thalamus, Putamen, and Caudate in Machado-Joseph Disease J Neuroimaging 2010 in press 10.1111/j.1552-6569.2010.00553.x 21122004

[B62] Klockgether T Skalej M Wedekind D Luft AR Welte D Schulz JB Abele M Burk K Laccone F Brice A Autosomal dominant cerebellar ataxia type I. MRI-based volumetry of posterior fossa structures and basal ganglia in spinocerebellar ataxia types 1, 2 and 3 Brain 1998 121 Pt 9 1687 1693 9762957 10.1093/brain/121.9.1687

[B63] D'Abreu A Franca M Appenzeller S Lopes-Cendes I Cendes F Axonal dysfunction in the deep white matter in Machado-Joseph disease J Neuroimaging 2009 19 1 9 12 10.1111/j.1552-6569.2008.00260.x 18482370

[B64] Takiyama Y Nishizawa M Tanaka H Kawashima S Sakamoto H Karube Y Shimazaki H Soutome M Endo K Ohta S The gene for Machado-Joseph disease maps to human chromosome 14q Nat Genet 1993 4 3 300 304 10.1038/ng0793-300 8358439

[B65] Kawaguchi Y Okamoto T Taniwaki M Aizawa M Inoue M Katayama S Kawakami H Nakamura S Nishimura M Akiguchi I CAG expansions in a novel gene for Machado-Joseph disease at chromosome 14q32.1 Nat Genet 1994 8 3 221 228 10.1038/ng1194-221 7874163

[B66] Ichikawa Y Goto J Hattori M Toyoda A Ishii K Jeong SY Hashida H Masuda N Ogata K Kasai F The genomic structure and expression of MJD, the Machado-Joseph disease gene J Hum Genet 2001 46 7 413 422 10.1007/s100380170060 11450850

[B67] Bettencourt C Santos C Montiel R Costa MC Cruz-Morales P Santos LR Simões N Kay T Vasconcelos J Maciel P Increased transcript diversity: novel splicing variants of Machado-Joseph Disease gene (*ATXN3*) Neurogenetics 2010 11 2 193 202 10.1007/s10048-009-0216-y 19714377

[B68] Takiyama Y Sakoe K Nakano I Nishizawa M Machado-Joseph disease: cerebellar ataxia and autonomic dysfunction in a patient with the shortest known expanded allele (56 CAG repeat units) of the MJD1 gene Neurology 1997 49 2 604 606 9270607 10.1212/wnl.49.2.604

[B69] Quan F Egan R DB J Popovich B An unusually small 55 repeat MJD1 CAG allele in a patient with Machado-Joseph disease [abstract] Am J Hum Genet 1997 61 A318 10.1159/000008218 11053973

[B70] van Schaik IN Jobsis GJ Vermeulen M Keizers H Bolhuis PA de Visser M Machado-Joseph disease presenting as severe asymmetric proximal neuropathy J Neurol Neurosurg Psychiatry 1997 63 4 534 536 10.1136/jnnp.63.4.534 9343141 PMC2169790

[B71] van Alfen N Sinke RJ Zwarts MJ Gabreels-Festen A Praamstra P Kremer BP Horstink MW Intermediate CAG repeat lengths (53,54) for MJD/SCA3 are associated with an abnormal phenotype Ann Neurol 2001 49 6 805 807 10.1002/ana.1089 11409435

[B72] Gu W Ma H Wang K Jin M Zhou Y Liu X Wang G Shen Y The shortest expanded allele of the MJD1 gene in a Chinese MJD kindred with autonomic dysfunction Eur Neurol 2004 52 2 107 111 10.1159/000080221 15316156

[B73] Padiath QS Srivastava AK Roy S Jain S Brahmachari SK Identification of a novel 45 repeat unstable allele associated with a disease phenotype at the MJD1/SCA3 locus Am J Med Genet B Neuropsychiatr Genet 2005 133B 1 124 126 10.1002/ajmg.b.30088 15457499

[B74] Walker FO Huntington's disease Lancet 2007 369 9557 218 228 10.1016/S0140-6736(07)60111-1 17240289

[B75] Goto J Watanabe M Ichikawa Y Yee SB Ihara N Endo K Igarashi S Takiyama Y Gaspar C Maciel P Machado-Joseph disease gene products carrying different carboxyl termini Neurosci Res 1997 28 4 373 377 10.1016/S0168-0102(97)00056-4 9274833

[B76] Alternative Splicing Prediction DataBase http://www.caspur.it/ASPicDB

[B77] Gales L Cortes L Almeida C Melo CV do Carmo Costa M Maciel P Clarke DT Damas AM Macedo-Ribeiro S Towards a structural understanding of the fibrillization pathway in Machado-Joseph's disease: trapping early oligomers of non-expanded ataxin-3 J Mol Biol 2005 353 3 642 654 10.1016/j.jmb.2005.08.061 16194547

[B78] Tzvetkov N Breuer P Josephin domain-containing proteins from a variety of species are active de-ubiquitination enzymes Biol Chem 2007 388 9 973 978 10.1515/BC.2007.107 17696782

[B79] Albrecht M Golatta M Wullner U Lengauer T Structural and functional analysis of ataxin-2 and ataxin-3 Eur J Biochem 2004 271 15 3155 3170 10.1111/j.1432-1033.2004.04245.x 15265035

[B80] Riess O Rub U Pastore A Bauer P Schols L SCA3: neurological features, pathogenesis and animal models Cerebellum 2008 7 2 125 137 10.1007/s12311-008-0013-4 18418689

[B81] Nijman SM Luna-Vargas MP Velds A Brummelkamp TR Dirac AM Sixma TK Bernards R A genomic and functional inventory of deubiquitinating enzymes Cell 2005 123 5 773 786 10.1016/j.cell.2005.11.007 16325574

[B82] Todi SV Winborn BJ Scaglione KM Blount JR Travis SM Paulson HL Ubiquitination directly enhances activity of the deubiquitinating enzyme ataxin-3 EMBO J 2009 28 4 372 382 10.1038/emboj.2008.289 19153604 PMC2646149

[B83] Ferro A Carvalho AL Teixeira-Castro A Almeida C Tome RJ Cortes L Rodrigues AJ Logarinho E Sequeiros J Macedo-Ribeiro S NEDD8: a new ataxin-3 interactor Biochim Biophys Acta 2007 1773 11 1619 1627 10.1016/j.bbamcr.2007.07.012 17935801

[B84] Li F Macfarlan T Pittman RN Chakravarti D Ataxin-3 is a histone-binding protein with two independent transcriptional corepressor activities J Biol Chem 2002 277 47 45004 45012 10.1074/jbc.M205259200 12297501

[B85] Burnett BG Pittman RN The polyglutamine neurodegenerative protein ataxin 3 regulates aggresome formation Proc Natl Acad Sci USA 2005 102 12 4330 4335 10.1073/pnas.0407252102 15767577 PMC555481

[B86] Rodrigues AJ do Carmo Costa M Silva TL Ferreira D Bajanca F Logarinho E Maciel P Absence of ataxin-3 leads to cytoskeletal disorganization and increased cell death Biochim Biophys Acta 1803 10 1154 1163 10.1016/j.bbamcr.2010.07.004 20637808

[B87] do Carmo Costa M Bajanca F Rodrigues AJ Tome RJ Corthals G Macedo-Ribeiro S Paulson HL Logarinho E Maciel P Ataxin-3 plays a role in mouse myogenic differentiation through regulation of integrin subunit levels PLoS One 5 7 e11728 10.1371/journal.pone.0011728 PMC2909204 20668528

[B88] Mauri PL Riva M Ambu D De Palma A Secundo F Benazzi L Valtorta M Tortora P Fusi P Ataxin-3 is subject to autolytic cleavage FEBS J 2006 273 18 4277 4286 10.1111/j.1742-4658.2006.05419.x 16939621

[B89] Paulson HL Perez MK Trottier Y Trojanowski JQ Subramony SH Das SS Vig P Mandel JL Fischbeck KH Pittman RN Intranuclear inclusions of expanded polyglutamine protein in spinocerebellar ataxia type 3 Neuron 1997 19 2 333 344 10.1016/S0896-6273(00)80943-5 9292723

[B90] Schmidt T Lindenberg KS Krebs A Schols L Laccone F Herms J Rechsteiner M Riess O Landwehrmeyer GB Protein surveillance machinery in brains with spinocerebellar ataxia type 3: redistribution and differential recruitment of 26S proteasome subunits and chaperones to neuronal intranuclear inclusions Ann Neurol 2002 51 3 302 310 10.1002/ana.10101 11891825

[B91] Evert BO Schelhaas J Fleischer H de Vos RA Brunt ER Stenzel W Klockgether T Wullner U Neuronal intranuclear inclusions, dysregulation of cytokine expression and cell death in spinocerebellar ataxia type 3 Clin Neuropathol 2006 25 6 272 281 17140157

[B92] Rub U de Vos RA Brunt ER Sebesteny T Schols L Auburger G Bohl J Ghebremedhin E Gierga K Seidel K Spinocerebellar ataxia type 3 (SCA3): thalamic neurodegeneration occurs independently from thalamic ataxin-3 immunopositive neuronal intranuclear inclusions Brain Pathol 2006 16 3 218 227 10.1111/j.1750-3639.2006.00022.x 16911479 PMC8095748

[B93] Gaspar C Lopes-Cendes I Hayes S Goto J Arvidsson K Dias A Silveira I Maciel P Coutinho P Lima M Ancestral origins of the Machado-Joseph disease mutation: a worldwide haplotype study Am J Hum Genet 2001 68 2 523 528 10.1086/318184 11133357 PMC1235286

[B94] Martins S Calafell F Gaspar C Wong VC Silveira I Nicholson GA Brunt ER Tranebjaerg L Stevanin G Hsieh M Asian origin for the worldwide-spread mutational event in Machado-Joseph disease Arch Neurol 2007 64 10 1502 1508 10.1001/archneur.64.10.1502 17923634

[B95] Lima M Doença de Machado-Joseph nos Açores: Estudo epidemiológico, biodemográfico e genético 1996 Ponta Delgada: University of the Azores

[B96] Lima M Mayer FM Coutinho P Abade A Origins of a mutation: population genetics of Machado-Joseph disease in the Azores (Portugal) Hum Biol 1998 70 6 1011 1023 9825593

[B97] Bettencourt C Silva-Fernandes A Montiel R Santos C Maciel P Lima M Santos C, Lima M Triplet Repeats: Features, Dynamics and Evolutionary Mechanisms Recent Advances in Molecular Biology and Evolution: Applications to Biological Anthropology 2007 Kerala: Research Signpost 83 114

[B98] Lima M Costa MC Montiel R Ferro A Santos C Silva C Bettencourt C Sousa A Sequeiros J Coutinho P Population genetics of wild-type CAG repeats in the Machado-Joseph disease gene in Portugal Hum Hered 2005 60 3 156 163 10.1159/000090035 16340213

[B99] Martins S Calafell F Wong VC Sequeiros J Amorim A A multistep mutation mechanism drives the evolution of the CAG repeat at MJD/SCA3 locus Eur J Hum Genet 2006 14 8 932 940 10.1038/sj.ejhg.5201643 16724006

[B100] Sequeiros J Análise genética da variação fenotípica na doença de Machado-Joseph 1989 PhD Dissertation. Instituto de Ciências Biomédicas de Abel Salazar, Universidade do Porto

[B101] Maciel P Gaspar C DeStefano AL Silveira I Coutinho P Radvany J Dawson DM Sudarsky L Guimaraes J Loureiro JE Correlation between CAG repeat length and clinical features in Machado-Joseph disease Am J Hum Genet 1995 57 1 54 61 7611296 PMC1801255

[B102] Maruyama H Nakamura S Matsuyama Z Sakai T Doyu M Sobue G Seto M Tsujihata M Oh-i T Nishio T Molecular features of the CAG repeats and clinical manifestation of Machado-Joseph disease Hum Mol Genet 1995 4 5 807 812 10.1093/hmg/4.5.807 7633439

[B103] Bettencourt C Santos C Montiel R Kay T Vasconcelos J Maciel P Lima M The (CAG)(n) tract of Machado-Joseph Disease gene (ATXN3): a comparison between DNA and mRNA in patients and controls Eur J Hum Genet 2009 10.1038/ejhg.2009.215 PMC2987309 19935829

[B104] Takiyama Y Igarashi S Rogaeva EA Endo K Rogaev EI Tanaka H Sherrington R Sanpei K Liang Y Saito M Evidence for inter-generational instability in the CAG repeat in the MJD1 gene and for conserved haplotypes at flanking markers amongst Japanese and Caucasian subjects with Machado-Joseph disease Hum Mol Genet 1995 4 7 1137 1146 10.1093/hmg/4.7.1137 8528200

[B105] Lerer I Merims D Abeliovich D Zlotogora J Gadoth N Machado-Joseph disease: correlation between the clinical features, the CAG repeat length and homozygosity for the mutation Eur J Hum Genet 1996 4 1 3 7 8800925 10.1159/000472162

[B106] Sobue G Doyu M Nakao N Shimada N Mitsuma T Maruyama H Kawakami S Nakamura S Homozygosity for Machado-Joseph disease gene enhances phenotypic severity J Neurol Neurosurg Psychiatry 1996 60 3 354 356 8609529 10.1136/jnnp.60.3.354-a PMC1073875

[B107] Tsuji S Molecular genetics of triplet repeats: unstable expansion of triplet repeats as a new mechanism for neurodegenerative diseases Intern Med 1997 36 1 3 8 10.2169/internalmedicine.36.3 9058092

[B108] Bettencourt C Fialho RN Santos C Montiel R Bruges-Armas J Maciel P Lima M Segregation distortion of wild-type alleles at the Machado-Joseph disease locus: a study in normal families from the Azores islands (Portugal) J Hum Genet 2008 53 4 333 339 10.1007/s10038-008-0261-7 18286225

[B109] Igarashi S Takiyama Y Cancel G Rogaeva EA Sasaki H Wakisaka A Zhou YX Takano H Endo K Sanpei K Intergenerational instability of the CAG repeat of the gene for Machado-Joseph disease (MJD1) is affected by the genotype of the normal chromosome: implications for the molecular mechanisms of the instability of the CAG repeat Hum Mol Genet 1996 5 7 923 932 10.1093/hmg/5.7.923 8817326

[B110] DeStefano AL Cupples LA Maciel P Gaspar C Radvany J Dawson DM Sudarsky L Corwin L Coutinho P MacLeod P A familial factor independent of CAG repeat length influences age at onset of Machado-Joseph disease Am J Hum Genet 1996 59 1 119 127 8659514 PMC1915115

[B111] van de Warrenburg BP Hendriks H Durr A van Zuijlen MC Stevanin G Camuzat A Sinke RJ Brice A Kremer BP Age at onset variance analysis in spinocerebellar ataxias: a study in a Dutch-French cohort Ann Neurol 2005 57 4 505 512 10.1002/ana.20424 15747371

[B112] Jardim L Silveira I Pereira ML do Ceu Moreira M Mendonca P Sequeiros J Giugliani R Searching for modulating effects of SCA2, SCA6 and DRPLA CAG tracts on the Machado-Joseph disease (SCA3) phenotype Acta Neurol Scand 2003 107 3 211 214 10.1034/j.1600-0404.2003.00046.x 12614315

[B113] Lopes-Cendes I Silveira I Maciel P Gaspar C Radvany J Chitayat D Babul R Stewart J Dolliver M Robitaille Y Limits of clinical assessment in the accurate diagnosis of Machado-Joseph disease Arch Neurol 1996 53 11 1168 1174 8912491 10.1001/archneur.1996.00550110120020

[B114] Sequeiros J Maciel P Taborda F Ledo S Rocha JC Lopes A Reto F Fortuna AM Rousseau M Fleming M Prenatal diagnosis of Machado-Joseph disease by direct mutation analysis Prenat Diagn 1998 18 6 611 617 10.1002/(SICI)1097-0223(199806)18:6<611::AID-PD289>3.0.CO;2-Y 9664608

[B115] Drusedau M Dreesen JC De Die-Smulders C Hardy K Bras M Dumoulin JC Evers JL Smeets HJ Geraedts JP Herbergs J Preimplantation genetic diagnosis of spinocerebellar ataxia 3 by (CAG)(n) repeat detection Mol Hum Reprod 2004 10 1 71 75 10.1093/molehr/gah008 14665709

[B116] Gonzalez C Lima M Kay T Silva C Santos C Santos J Short-term psychological impact of predictive testing for Machado-Joseph disease: depression and anxiety levels in individuals at risk from the Azores (Portugal) Community Genet 2004 7 4 196 201 10.1159/000082262 15692194

[B117] Paul C Martin I do Rosario Silva M Silva M Coutinho P Sequeiros J Living with Machado-Joseph disease in a small rural community of the Tagus valley Community Genet 1999 2 4 190 195 10.1159/000016211 14960841

[B118] Sequeiros J Sequeiros J General Protocol of the National Program of Predictive Testing and Genetic Counselling in Machado-Joseph disease Predictive Testing in Machado-Joseph Disease (in Portuguese) 1996 Porto: UnIGENe-IBMC

[B119] Lima M Kay T Vasconcelos J Mota-Vieira L Gonzalez C Peixoto A Abade A MacLeod P Graca R Santos J Disease knowledge and attitudes toward predictive testing and prenatal diagnosis in families with Machado-Joseph disease from the Azores Islands (Portugal) Community Genet 2001 4 1 36 42 10.1159/000051154 11493751

[B120] Leite A Paúl C Sequeiros J O bem-estar psicológico em individuos de risco para doenças neurológicas hereditárias de aparecimento tardio e controlos [article in Portuguese] Psicologia, Saúde & Doença 2002 3 4 113 11821044493

[B121] Rolim L Leite A Ledo S Paneque M Sequeiros J Fleming M Psychological aspects of pre-symptomatic testing for Machado-Joseph disease and familial amyloid polyneuropathy type I Clin Genet 2006 69 4 297 305 10.1111/j.1399-0004.2006.00606.x 16630162

[B122] Abe K Itoyama Y Psychological consequences of genetic testing for spinocerebellar ataxia in the Japanese European Journal of Neurology 1997 4 593 600 10.1111/j.1468-1331.1997.tb00411.x

[B123] Smith CO Lipe HP Bird TD Impact of presymptomatic genetic testing for hereditary ataxia and neuromuscular disorders Arch Neurol 2004 61 6 875 880 10.1001/archneur.61.6.875 15210524

[B124] Cancel G Gourfinkel-An I Stevanin G Didierjean O Abbas N Hirsch E Agid Y Brice A Somatic mosaicism of the CAG repeat expansion in spinocerebellar ataxia type 3/Machado-Joseph disease Hum Mutat 1998 11 1 23 27 9450899 10.1002/(SICI)1098-1004(1998)11:1<23::AID-HUMU4>3.0.CO;2-M

[B125] Ogawa M Pharmacological treatments of cerebellar ataxia Cerebellum 2004 3 2 107 111 10.1080/147342204100032331 15233578

[B126] Correia M Coutinho P Silva MC Guimaraes J Amado J Matos E Evaluation of the effect of sulphametoxazole and trimethoprim in patients with Machado-Joseph disease Rev Neurol 1995 23 121 632 634 8597984

[B127] Tuite PJ Rogaeva EA St George-Hyslop PH Lang AE Dopa-responsive parkinsonism phenotype of Machado-Joseph disease: confirmation of 14q CAG expansion Ann Neurol 1995 38 4 684 687 10.1002/ana.410380422 7574470

[B128] Buhmann C Bussopulos A Oechsner M Dopaminergic response in Parkinsonian phenotype of Machado-Joseph disease Mov Disord 2003 18 2 219 221 10.1002/mds.10322 12539220

[B129] Wilder-Smith E Tan EK Law HY Zhao Y Ng I Wong MC Spinocerebellar ataxia type 3 presenting as an L-DOPA responsive dystonia phenotype in a Chinese family J Neurol Sci 2003 213 1-2 25 28 10.1016/S0022-510X(03)00129-1 12873751

[B130] Nandagopal R Moorthy SG Dramatic levodopa responsiveness of dystonia in a sporadic case of spinocerebellar ataxia type 3 Postgrad Med J 2004 80 944 363 365 10.1136/pgmj.2003.015297 15192175 PMC1743016

[B131] Kanai K Kuwabara S Arai K Sung JY Ogawara K Hattori T Muscle cramp in Machado-Joseph disease: altered motor axonal excitability properties and mexiletine treatment Brain 2003 126 Pt 4 965 973 12615652 10.1093/brain/awg073

[B132] Mello KA Abbott BP Effect of sulfamethoxazole and trimethoprim on neurologic dysfunction in a patient with Joseph's disease Arch Neurol 1988 45 2 210 213 3257688 10.1001/archneur.1988.00520260098028

[B133] Azulay JP Blin O Mestre D Sangla I Serratrice G Contrast sensitivity improvement with sulfamethoxazole and trimethoprim in a patient with Machado-Joseph disease without spasticity J Neurol Sci 1994 123 1-2 95 99 10.1016/0022-510X(94)90209-7 8064328

[B134] Sakai T Matsuishi T Yamada S Komori H Iwashita H Sulfamethoxazole-trimethoprim double-blind, placebo-controlled, crossover trial in Machado-Joseph disease: sulfamethoxazole-trimethoprim increases cerebrospinal fluid level of biopterin J Neural Transm Gen Sect 1995 102 2 159 172 10.1007/BF01276511 8748680

[B135] Schulte T Mattern R Berger K Szymanski S Klotz P Kraus PH Przuntek H Schols L Double-blind crossover trial of trimethoprim-sulfamethoxazole in spinocerebellar ataxia type 3/Machado-Joseph disease Arch Neurol 2001 58 9 1451 1457 10.1001/archneur.58.9.1451 11559318

[B136] Monte TL Rieder CR Tort AB Rockenback I Pereira ML Silveira I Ferro A Sequeiros J Jardim LB Use of fluoxetine for treatment of Machado-Joseph disease: an open-label study Acta Neurol Scand 2003 107 3 207 210 10.1034/j.1600-0404.2003.02132.x 12614314

[B137] Shirasaki H Ishida C Nakajima T Kamei H Koide T Fukuhara N [A quantitative evaluation of spinocerebellar degeneration by an acoustic analysis--the effect of taltirelin hydrate on patients with Machado-Joseph disease] Rinsho Shinkeigaku 2003 43 4 143 148 12884823

[B138] Takei A Fukazawa T Hamada T Sohma H Yabe I Sasaki H Tashiro K Effects of tandospirone on "5-HT1A receptor-associated symptoms" in patients with Machado-Josephe disease: an open-label study Clin Neuropharmacol 2004 27 1 9 13 10.1097/00002826-200401000-00005 15090930

[B139] Liu CS Hsu HM Cheng WL Hsieh M Clinical and molecular events in patients with Machado-Joseph disease under lamotrigine therapy Acta Neurol Scand 2005 111 6 385 390 10.1111/j.1600-0404.2005.00405.x 15876340

[B140] Underwood BR Rubinsztein DC Spinocerebellar ataxias caused by polyglutamine expansions: a review of therapeutic strategies Cerebellum 2008 7 2 215 221 10.1007/s12311-008-0026-z 18418676

[B141] D'Abreu A Franca MC Paulson HL Lopes-Cendes I Caring for Machado-Joseph disease: current understanding and how to help patients Parkinsonism Relat Disord 2010 16 1 2 7 10.1016/j.parkreldis.2009.08.012 19811945 PMC2818316

[B142] Schmitt I Linden M Khazneh H Evert BO Breuer P Klockgether T Wuellner U Inactivation of the mouse Atxn3 (ataxin-3) gene increases protein ubiquitination Biochem Biophys Res Commun 2007 362 3 734 739 10.1016/j.bbrc.2007.08.062 17764659

[B143] Rodrigues AJ Coppola G Santos C Costa Mdo C Ailion M Sequeiros J Geschwind DH Maciel P Functional genomics and biochemical characterization of the C. elegans orthologue of the Machado-Joseph disease protein ataxin-3 FASEB J 2007 21 4 1126 1136 10.1096/fj.06-7002com 17234717

[B144] Miller VM Xia H Marrs GL Gouvion CM Lee G Davidson BL Paulson HL Allele-specific silencing of dominant disease genes Proc Natl Acad Sci USA 2003 100 12 7195 7200 10.1073/pnas.1231012100 12782788 PMC165852

[B145] Alves S Nascimento-Ferreira I Auregan G Hassig R Dufour N Brouillet E Pedroso de Lima MC Hantraye P Pereira de Almeida L Deglon N Allele-specific RNA silencing of mutant ataxin-3 mediates neuroprotection in a rat model of Machado-Joseph disease PLoS One 2008 3 10 e3341 10.1371/journal.pone.0003341 18841197 PMC2553199

[B146] Hu J Matsui M Gagnon KT Schwartz JC Gabillet S Arar K Wu J Bezprozvanny I Corey DR Allele-specific silencing of mutant huntingtin and ataxin-3 genes by targeting expanded CAG repeats in mRNAs Nat Biotechnol 2009 27 5 478 484 10.1038/nbt.1539 19412185 PMC2765218

